# The Influence of Age and Oral Health on Taste Perception in Older Adults: A Case-Control Study

**DOI:** 10.3390/nu13114166

**Published:** 2021-11-21

**Authors:** Sonila Alia, Luca Aquilanti, Sofia Pugnaloni, Alice Di Paolo, Giorgio Rappelli, Arianna Vignini

**Affiliations:** 1 Department of Clinical Specialistic and Dental Sciences, Università Politecnica delle Marche, Via Tronto 10, 60126 Ancona, Italy; s.alia@pm.univpm.it (S.A.); l.aquilanti@pm.univpm.it (L.A.); s.pugnaloni@pm.univpm.it (S.P.); a.dipaolo@pm.univpm.it (A.D.P.); g.rappelli@staff.univpm.it (G.R.); 2 Dentistry Clinic, National Institute of Health and Science of Aging, IRCCS INRCA, Via Tronto 10, 60126 Ancona, Italy

**Keywords:** nutritional status, taste perception, oral health, masticatory performance

## Abstract

Declining gustatory function, nutrition, and oral health are important elements of health in older adults that can affect the aging process. The aim of the present work was to investigate the effect of age and oral status on taste discrimination in two different groups of elderly subjects living either in an Italian residential institution (TG) or in the community (CG). A total of 90 subjects were enrolled in the study (58 CG vs. 32 TG). Masticatory performance (MP) was assessed using the two-color mixing ability test. Taste function was evaluated using cotton pads soaked with six taste stimuli (salty, acid, sweet, bitter, fat and water). A positive correlation between age and missing teeth (r = 0.51, C.I. [0.33; 0.65], *p* < 0.0001), and a negative correlation between age and MP (r = −0.39, C.I. [−0.56; −0.20], *p* < 0.001) were found. Moreover, significant differences for salty taste, between TG and CG were detected (*p* < 0.05). Significant differences in bitter taste sensitivity between subjects wearing removable and non-removable prosthesis were also determined (*p* < 0.05). In addition, significant gender differences and between males in TG and CG were identified (*p* < 0.05). The best understanding of the relationship between MP, taste sensitivity, and nutritional factors is a necessary criterion for the development of new therapeutic strategies to address more effectively the problems associated with malnutrition in elderly subjects.

## 1. Introduction

Aging is a progressive, intrinsic, and universal process that occurs in every living being as the result of the interaction between individual’s genetics and the environment [1]. The great socio-economic development of the last century, medical advances, a better lifestyle, and fertility rate decrease have led to an increase in life expectancy. The World Health Organization (WHO) has calculated demographic projections showing an increase in the population over 65. These predictions will have a significant impact on the delivery of general and oral health care and treatment strategies within the geriatric patient population [2].

Oral health is an integral part of general health and affects the quality of life of an individual [3]. Poor oral health status is considered to be a strong predictor of the onset of adverse clinical outcomes, including mortality, among the community dwelling elderly [4]. The maintenance of a healthy mouth is crucial, since the worsening of oral health status leads to functional loss [5]. With regard to poor functional capacity, it has been shown that masticatory performance (MP) decreases with the loss of dental elements. In particular, at least ten pairs of occluding teeth are necessary for adequate chewing capacity [6]. Effective prosthetic solutions have been proposed to restore dentition and oral function with a consequent greater subjective appreciation. Unfortunately, prosthetic rehabilitation does not restore chewing performance in its totality and cannot be compared to natural dentition. Therefore, preventive strategies aimed at maintaining a good oral health status are necessary to prevent oral hypofunction [7].

The loss of masticatory function is responsible for diet restriction and impaired bolus formation, leading to two main consequences. First of all, it can cause interference with digestion and nutrient extraction. Secondly, the exclusion of some basic food (e.g., meat, fruit and vegetables), can lead to the exclusion of foods considered difficult to chew in favor of soft, easily chewed foods (e.g., refined carbohydrates and fats), inducing bad dietary practices and poor nutritional intakes [8]. As stated in previous studies, malnourishment is prevalent in the elderly population, especially in elderly hospitalized patients [9,10]. Overall, the nutritional status is one of the most important modifiable factors capable of affecting the health, the well-being, and the overall QoL of an individual [11].

The effects of physiological aging on the perception of taste are represented by the alterations of taste cells, the reduction of salivary production, and inability to fully chew food. Taste alterations can be classified as qualitative, which include dysgeusia (e.g., an alteration of taste sensitivity for foods previously enjoyed and which later become unpleasant) and quantitative, which consist of ageusia (total deficit), hypogeusia, and hypergeusia, a decrease or increase in taste sensitivity, respectively [12]. Several geriatric pathological conditions could lead to dysgeusia or ageusia. Among older adults, taste loss is frequently caused by multiple factors, including physiological changes such as impairment in taste receptor cells, poor oral health condition, and a deteriorating olfactory function. In addition, it is worsened by events related to aging such as poor general health, polypharmacy, and systemic diseases. The existence of taste disorders is commonly observed in elderly hospitalized patients for acute conditions, and it is often associated with poor oral hygiene and infections [13,14].

Researchers are still debating the severity of the taste loss that older adults daily experience. Overall, sour and bitter tastes seem to be the most impaired ones. Several studies report an age-related decline in the perception of also salty and sweet flavors, with contradictory results, while other papers are in agreement with an increased perception of umami taste [15]. There is still no consensus whether the physiological changes in taste perception could affect food preferences among old people, even though many authors found them less interested in sour tastes and pungent flavors [16].

Thus, the present study aimed at (i) verifying the impact of oral health on the nutritional status of institutionalized elderly subjects compared to home-living ones; (ii) analyzing the taste discernment by the administration of taste stimuli in all enrolled subjects; (iii) assessing any association between taste perception and presence of removable dentures. Gender differences were also explored.

## 2. Materials and Methods

The present cross-sectional study included 90 adults, 65 years old and over, living either at home (control group—CG) or in a residential aged care facility (test group—TG). 58 patients (CG) in outpatient medical treatment were recruited at the Dental Clinic of Università Politecnica delle Marche, Ancona, Italy, during the period April 2017–December 2017, and 32 patients (TG), living at “Casa di Riposo Grimani Buttari”, Osimo, Italy, participated in a comprehensive geriatric health examination from December 2018 to May 2019. The study was performed in accordance with the principles of the Declaration of Helsinki as revised in 2013 and was approved by the Institutional Review Board of Dentistry Clinic, Università Politecnica delle Marche, Ancona, Italy (ODO-EXP-107/18, 19 June 2018). Written informed consent was obtained from all enrolled subjects after the procedures had been fully explained.

Individual sociodemographic data and general health information were recorded for all participants. All included subjects had to be ≥65 years old and compliant. Subjects were excluded if they were <65 years, suffered from neurodegenerative conditions (e.g., Alzheimer, Parkinson, Dementia), had a diagnosis of diseases and disorders affecting the muscular system, had oro-facial pain, or were not compliant. The Institute’s healthcare team selected participants on the basis of subjects’ medical history. All the enrolled subjects underwent dental examination in which the number of retained teeth, the number of missing teeth, and the number of occlusal tooth unit were recorded. Moreover, the presence or lack of dental prostheses was noted. In addition, a masticatory test was carried out, using the two-color mixing test as described elsewhere [17]. Briefly, the test consists in the chewing of two-colored chewing gums (Hue-check Gum^®^, Orophys GmbH, Muri bei Bern, Switzerland). Each sample was chewed for 20 chewing cycles, as this number of strokes allows the assessment of MP. Boluses were collected, inserted between two sheets of transparent plastic, yielding samples of 1 ± 0.1 mm of thickness. Standardized photos were taken from both sides of each bolus, and all the obtained images were processed by computer, analyzing the measure of the area of pixels of different colors using the K-means clustering method [18]. At the end of the analysis, the software revealed the ratio between mixed and unmixed areas of the boluses, discriminating between the different MPs of the subjects.

The taste test was based on filter paper strips as described by Landis et al. [19] and modified as reported elsewhere [20,21]. Briefly, cotton pads, soaked with four substances (sodium chloride, citric acid, sucrose, and quinine hydrochloride) were applied to the protruded tongue, immediately posterior to its first third, either to the left or right side, in order to study lateralization too; each basic taste quality (salty, sour, sweet, and bitter) was presented at 4 different concentrations (Table 1).

In addition, pure rapeseed oil and water were administered to evoke fat and neutral taste, respectively. Rapeseed oil is a neutral oil which has a pale-yellow color and is almost odorless; the rapeseed oil was chosen instead of olive oil since this latter has a specific texture and increased volatility in the oral cavity, making it easily recognizable. Distilled water was used as a solvent, and taste solutions were freshly prepared on the morning of each testing session. Since gustatory stimulation also causes the activation of other sensory system (e.g., touch receptors), the test was performed so as to minimize the activation of other receptors. Subjects were required to wash their mouth with deionized water between samples to avoid carryover effects. Administration was randomized for the four concentrations, and the side of presentation was alternated: 36 cotton pads (18 for the left side and 18 for the right side) were used. The enrolled subjects had to identify the taste by choosing from a list that included eight descriptions: sweet, salty, bitter, sour, water, fat, nothing, I do not know (forced multiple choice). The test took about 20 min.

Data were analyzed using R statistical software (R Foundation for Statistical Computing, Vienna, Austria). The normal distribution of continuous variables was tested by the Kolmogorov-Smirnov test. For continuous data, Mann-Whitney test and t-student with Welch correction test were used. Chi-square test was used for significance of associations with categorical variables. Pearson correlation coefficient was used to assess correlations between the tested variables. The dichotomous dependent variable, taste stimuli perception, was introduced in a multiple logistic regression model to estimate its variation according to the independent variables and to verify the presence of possible confounders. Data were expressed as Mean ± SD. A value of *p* < 0.05 was considered statistically significant.

## 3. Results

Out of a total of 190 subjects attending “Casa di Riposo Grimani Buttari”, Osimo, Italy, only 32 (16.8%) subjects (8 males and 24 females) (TG) met the inclusion criteria and were enrolled in this study. The CG comprises 58 subjects (32 males and 26 females) in outpatient medical treatment recruited at the Dentistry Clinic of Università Politecnica delle Marche, Ancona, Italy. The CG group mean age was 74.6 ± 4.8, while the TG one was 86.4 ± 7.0 years (*p* < 0.001). In the CG, a mean loss of 5.3 ± 4.7 teeth was recorded, while in the TG it was 19.3 ± 9.1, *p* < 0.001. Table 2 shows oral health related data of the enrolled subjects.

The MP test results were analyzed in relation to the number of missing teeth, both in the CG and in the TG. The test showed a negative correlation between MP and missing teeth both in the TG and in the CG, r = −0.87, C.I. [−0.94; −0.76], *p* < 0.001, and r = −0.51. C.I. [−0.68; −0.28], *p* < 0.001, respectively. Overall, a negative correlation was showed between MP and missing teeth, r = −0.77, C.I. [−0.84; −0.66], *p* < 0.001. Furthermore, when correlating age with the number of missing teeth, a positive relationship was found (r = 0.51, C.I. [0.33; 0.65], *p* < 0.001). Conversely, when comparing age with MP, a negative correlation was found (r = −0.39, C.I. [−0.56; −0.20], *p* < 0.001). The multiple logistic regression model showed no influence of the statistically different characteristics of the study groups in the perception of taste stimuli (*p* > 0.05).

When assessing taste perception, the number of correct answers in the CG and in the TG were analyzed. Table 3 summarizes the results of the taste perception test in the two study groups, also pointing out correct answers for males and females within CG and TG.

According to the different types of stimuli, we found that only the salty taste perception was significantly different between the two groups (*p* < 0.05). In particular, the CG had a better taste performance than the TG. Furthermore, the comparison between the taste perceived by the wearers of removable and non-removable prosthesis was performed. In this case, only the bitter taste was statistically significant between the two groups (*p* < 0.05). In particular, those who did not formerly wear a dental prosthesis had a better taste perception performance than removable prostheses wearers. Finally, gender differences were analyzed in order to evaluate alterations in the perception of all tastes (Table 4). Bitter, sour, and sweet tastes were better recognized by females than males (*p* < 0.05).

## 4. Discussion

In the present study, performed on 32 older adults living in an Italian residential aged care facility and 58 autonomous controls, data relating to oral health status, MP, as well as taste perception were analyzed. A relationship between the number of missing teeth and the MP, and impairment in taste perception between CG and TG, between removable and non-removable prostheses wearers, and between genders were found.

In accordance with the literature, and with our previous work, in the present paper we found that as the number of missing teeth increases, the MP decreases [22,23,24]. Since in the CG the number of missing teeth was overall smaller than that of the TG, the MP was higher in first group of subjects than in the second one. An explanation of this could lie in the fact that tooth loss is associated with the aging process, and being progressive and cumulative, is more likely to diagnose both partial and total edentulism in the oldest age classes. Tooth loss leads to changes in eating habits, commonly attributed also to changes in the hedonistic quality of food [25,26]. With advancing age, it is more frequent that older adults present, even simultaneously, exhibit a low masticatory function and poor appetite [27]. Good oral health is essential for an adequate nutritional status, because the maintenance of a natural dentition can ensure an adequate masticatory function related to a balanced diet, that in turn affect the QoL in the elderly [28].

Aging is characterized by a reduction in overall sensory perception. The decline in taste and smell can lead to poor appetite and malnutrition, improper food choices, and inadequate nutrient intake [29]. Poor oral health can determine the onset of malnourishment, and the latter can in turn affect oral health. Indeed, insufficient nutrient consumption was associated with a reduction of muscle strength and physical capacity, and, as in a vicious circle, old age can increase the risk of low nutritional intake. In the last decades, several studies investigated the changes in taste perception that occur with aging with inconsistent results [30,31,32]. In the present paper, although in some cases no statistically significant differences were found between groups, CG was able to recognize a greater number of taste stimuli than TG, except for sweet taste that was perceived in the same proportion by both groups. Our findings are in accordance with previous studies, as most of them reported that older subjects required a higher concentration of primary tastes than young people [33,34]. However, the decrease in taste perception is not the same for all taste stimuli; in fact, if it becomes difficult to perceive bitterness, the ability to recognize the sweet taste is kept even in advanced age, with a consequent liking of sweet and high-calorie meals.

The reported statistically significant decrease in salty taste between the two analyzed groups may be related to the fact that in Italy the habitual salt intake is well above the recommended amounts [35]. The CG was composed by autonomous subjects, and they were able to prepare meals by their own. They used more salt in food than the TG, which on the contrary eats what is prepared by the cooks of the aged care facility. The cooks of the aged care facility reduce dietary salt intake to lower blood pressure and prevent deaths due to stroke or cardiovascular disease. Our findings may indicate that individuals who are living at home may be at risk for injuring their health by involuntary over-intake of a salty diet. On the other hand, institutionalization of older adults can also lead to nutritional deficiency if nutrient intake is not well managed, or taste sensation issues are not properly evaluated.

When comparing taste perception between subjects with and without denture prosthesis, removable prostheses wearers were less sensitive to the recognition of overall taste stimuli than those without prosthesis. These results are in accordance with those by da Silva et al. that showed that the presence of the denture is able to change the recognition of taste when compared to its absence, mainly for the bitter taste [36]. Our findings are also in agreement with data present in the literature, according to which the taste sensitivity is greatly reduced in people wearing dentures [37]. Conversely, some reports have shown that prosthetic carriers have an increased threshold to detect the sweet in solid foods [38]. This can be explained by the fact that prosthetic carriers have a reduced ability to grind food and a reduced rate of salivary secretion. In this context, sweet substances dissolve in a non-optimal way and thus reach the taste cells less easily.

The results showed gender differences in the ability to recognize different tastes, showing that on average females are more sensitive in the recognition of all tastes (except fat, *p* > 0.05).

Overall, in the average of the results between males and females, only bitter and sweet tastes reached statistical significance. These results provide new insights into the identification of the taste sensitivity related to gender. In general, gender differences in taste-related behaviors are associated with circulating estrogen levels, which can modulate the detectability and preference of taste. Hormones, in fact, can modulate the responses to the taste stimuli, performing both organizational and activation roles in the regulation of gustatory responses [39]. Regardless of estrogen status, peripheral taste perception differs between males and females, indicating that the same taste stimuli produce differential inputs in the brains of males and females. Mechanisms contributing to differences in gustatory processing, and the extent to which male and female gustatory function vary, are awaiting further clarification, which should include the analysis of electrophysiological responses caused by taste in each of the gustatory nerves. A previous study attempted to analyze the differentiation of the spatial distribution of different taste perception frequencies between the two genders, through the use of magnetoencephalography (MEG) [40]. Thanks to this neuroimaging technique it was possible to deduce that females show more channels with high frequencies due to the stimulation with the sweet and bitter taste, guaranteeing a better gustatory response. On the contrary, as for the salty taste, they show channels with low frequencies and, consequently, a lesser gustatory response.

Beside this, gender differences may be due to the fact that females have higher papillae density (FPD) than males, in accordance with literature [41]. Furthermore, a regular reducing of FPD is observed with age, an effect more evident in males than in females, thus confirming males higher susceptibility to FPD lowering with age [42], in agreement with the present results.

In our study, we also analyzed differences between male and female within TG and CG. Remarkably, only males showed alterations inside the two groups, with CG more able to recognize tastes than TG. The data herein presented thus show the interplay of gender and age in defining interindividual variations in gustatory responsiveness.

The present report has some limitations that provide opportunities for future studies. The small number of the enrolled subjects living in the residential aged care facility restricted the sample of this study, due to the non-compliance of most of the residents. The cross-sectional design of the study provided only a picture of the situation, making it difficult to generalize our results to the whole older adult population. Regardless, cross-sectional studies are a useful tool for establishing preliminary evidence in planning future research that should aim at enlarging the sample size and deeply investigating the relationship among taste stimuli perception, age, and oral health status in older adults. Finally, the statistically significant differences between CG and TG and other confounding factors, that might not have been accounted for, may have influenced the results of the study. Although the results of this study should be interpreted with caution, multiple logistic regression models showed no influence of the statistically different characteristics of the study groups in the perception of taste stimuli. The best understanding of the relationship between masticatory performance, taste sensitivity, and nutritional factors is a necessary prerequisite for the development of new therapeutic strategies to more effectively address the problems associated with malnutrition of the geriatric patient. In light of these considerations, the importance of continuous training of operators in the field of oral health of elderly patients is warranted in order to implement treatment plans aimed at preserving natural teeth.

## 5. Conclusions

Within the limitation of this study, the analyses carried out allow us to confirm that masticatory performance is associated with the number of missing teeth. In particular, masticatory performance is statistically significantly lower in older adults living in a residential aged care facility compared to autonomous subjects, as a result of the fewer number of retained teeth and their poorer oral health status.

Taste results depict a complex interplay of different factors affecting gustatory acuity, among which oral health, age and sex may have a role. Though the association between perception and food intake still requires a more comprehensive analysis, the present data may be important for precision nutrition, as they support the hypothesis that inter-individual differences in taste perception must be taken into account so as to better understand food preferences and food intake and so achieve a tailored adherence to nutritional recommendations.

## Figures and Tables

**Table 1:** Concentrations of taste stimuli.

Stimulus	Substance	Concentration
Sweetness	Sucrose	0.05 g/mL
		0.1 g/mL
		0.2 g/mL
		0.5 g/mL
Saltiness	Sodium Chloride	0.016 g/mL
		0.04 g/mL
		0.1 g/mL
		0.25 g/mL
Bitterness	Quinine	0.0004 g/mL
		0.0009 g/mL
		0.0024 g/mL
		0.006 g/mL
Sourness	Citric Acid	0.05 g/mL
		0.09 g/mL
		0.165 g/mL
		0.3 g/ml
Fat	Rapeseed oil	Pure
Neutral	Deionized water	Pure

**Table 2:** Sociodemographic and Oral health related data of the studied groups. Data are expressed as Mean ± Standard Deviation.

	Control Group	Test Group	*p*-Value
Age (years)	74.6 ± 4.8	86.4 ± 7.0	<0.001
Sex (M/F)	32/26	8/24	
Height (cm)	162.9 ± 8.5	154.1 ± 9.9	<0.001
Weight (Kg)	74.9 ± 15.1	63.8 ± 11.8	<0.001
BMI (Kg/m^2^)	28.1 ± 4.6	27.0 ± 5.2	NS ^2^
No. of drugs	3.7 ± 2.3	8.2 ± 3.2	<0.001
Missing Teeth	5.3 ± 4.7	19.3 ± 9.1	<0.001
Occlusal Units	10.9 ± 2.5	3.6 ± 4.4	<0.001
DMFT ^1^	13.3 ± 5.4	20.1 ± 8.3	<0.01
Masticatory Performance	0.43 ± 0.17	0.23 ± 0.18	<0.001

^1^ Decayed Missing Filled Teeth; ^2^ Not statistically significant.

**Table 3:** Taste stimuli test: percentage of correct answer in the study groups.

Stimuli	Control Group (CG)	Test Group (TG)	*p*-Value	Males		*p*-Value	Females		*p*-Value
				CG	TG		CG	TG	
Sweetness Sucrose (0.05 g/mL)	22.4%	31.3%	NS ^1^	19.4%	37.5%	NS ^1^	26.1%	29.2%	NS ^1^
Sweetness Sucrose (0.1 g/mL)	44.8%	50.0%	NS ^1^	36.1%	25.0%	NS ^1^	56.5%	58.3%	NS ^1^
Sweetness Sucrose (0.2 g/mL)	56.9%	53.1%	NS ^1^	52.8%	37.5%	NS ^1^	60.9%	58.3%	NS ^1^
Sweetness Sucrose (0.5 g/mL)	63.8%	50.0%	NS ^1^	52.8%	25.0%	NS ^1^	78.3%	58.3%	NS ^1^
Saltiness Sodium Chloride (0.016 g/mL)	19.1%	12.5%	NS ^1^	19.4%	0.0%	<0.01	17.4%	16.7%	NS ^1^
Saltiness Sodium Chloride (0.04 g/mL)	46.6%	34.4%	NS ^1^	38.9%	37.5%	NS ^1^	56.5%	33.3%	NS ^1^
Saltiness Sodium Chloride (0.1 g/mL)	53.5%	34.4%	NS ^1^	50.0%	0.0%	<0.0001	56.5%	45.8%	NS ^1^
Saltiness Sodium Chloride (0.25 g/mL)	55.2%	34.4%	<0.05	50.0%	0.0%	<0.0001	60.9%	45.8%	NS ^1^
Bitterness Quinine (0.0004 g/mL)	24.1%	18.8%	NS ^1^	19.4%	0.0%	<0.001	30.4%	25.0%	NS ^1^
Bitterness Quinine (0.0009 g/mL)	53.5%	40.6%	NS ^1^	41.7%	25.0%	NS ^1^	69.6%	45.8%	NS ^1^
Bitterness Quinine (0.0024 g/mL)	58.6%	62.5%	NS ^1^	55.6%	50.0%	NS ^1^	60.9%	66.7%	NS ^1^
Bitterness Quinine (0.006 g/mL)	77.9%	59.4%	NS ^1^	66.7%	62.5%	NS ^1^	91.3%	58.3%	<0.01
Sourness Citric Acid (0.05 g/mL)	37.9%	31.3%	NS ^1^	25.0%	12.5%	NS ^1^	56.5%	37.5%	NS ^1^
Sourness Citric Acid (0.09 g/mL)	53.5%	34.4%	NS ^1^	47.2%	12.5%	<0.05	60.9%	41.7%	NS ^1^
Sourness Citric Acid (0.165 g/mL)	50.0%	50.0%	NS ^1^	50.0%	12.5%	<0.05	56.5%	62.5%	NS ^1^
Sourness Citric Acid (0.3 g/mL)	58.6%	46.9%	NS ^1^	55.6%	25.0%	NS ^1^	60.9%	54.2%	NS ^1^
Fat Rapeseed Oil	17.2%	9.4%	NS ^1^	19.4%	0.0%	<0.01	13.0%	12.5%	NS ^1^
Neutral Deionized Water	13.8%	9.4%	NS ^1^	13.9%	0.0%	<0.05	13.0%	12.5%	NS ^1^

^1^ Not statistically significant.

**Table 4:** Taste stimuli test: percentage of correct answer in the study groups.

Stimuli	Control Group		*p*-Value	Test Group		*p*-Value
	Male	Female		Male	Female	
Sweetness Sucrose (0.05 g/mL)	19.4%	26.1%	NS ^1^	37.5%	29.2%	NS ^1^
Sweetness Sucrose (0.1 g/mL)	36.1%	56.5%	NS ^1^	25.0%	58.3%	NS ^1^
Sweetness Sucrose (0.2 g/mL)	52.8%	60.9%	NS ^1^	37.5%	58.3%	NS ^1^
Sweetness Sucrose (0.5 g/mL)	52.8%	78.3%	<0.05	25.0%	58.3%	NS ^1^
Saltiness Sodium Chloride (0.016 g/mL)	19.4%	17.4%	NS ^1^	0.0%	16.7%	<0.05
Saltiness Sodium Chloride (0.04 g/mL)	38.9%	56.5%	NS ^1^	37.5%	33.3%	NS ^1^
Saltiness Sodium Chloride (0.1 g/mL)	50.0%	56.5%	NS ^1^	0.0%	45.8%	<0.001
Saltiness Sodium Chloride (0.25 g/mL)	50.0%	60.9%	NS ^1^	0.0%	45.8%	<0.001
Bitterness Quinine (0.0004 g/mL)	19.4%	30.4%	NS ^1^	0.0%	25.0%	<0.05
Bitterness Quinine (0.0009 g/mL)	41.7%	69.6%	<0.05	25.0%	45.8%	NS ^1^
Bitterness Quinine (0.0024 g/mL)	55.6%	60.9%	NS ^1^	50.0%	66.7%	NS ^1^
Bitterness Quinine (0.006 g/mL)	66.7%	91.3%	<0.05	62.5%	58.3%	NS ^1^
Sourness Citric Acid (0.05 g/mL)	25.0%	56.5%	<0.05	12.5%	37.5%	NS ^1^
Sourness Citric Acid (0.09 g/mL)	47.2%	60.9%	NS ^1^	12.5%	41.7%	NS ^1^
Sourness Citric Acid (0.165 g/mL)	50.0%	56.5%	NS ^1^	12.5%	62.5%	<0.01
Sourness Citric Acid (0.3 g/mL)	55.6%	60.9%	NS ^1^	25.0%	54.2%	NS ^1^
Fat Rapeseed Oil	19.4%	13.0%	NS ^1^	0.0%	12.5%	NS ^1^
Neutral Deionized Water	13.9%	13.0%	NS ^1^	0.0%	12.5%	NS ^1^

^1^ Not statistically significant.

## Data Availability

The data sets generated and/or analysed during the present study are available from the corresponding author on reasonable request.
